# Implementing a Decommissioning Programme in Swedish Healthcare: Experiences of Healthcare Managers

**DOI:** 10.1177/11786329241299316

**Published:** 2024-11-19

**Authors:** Inga-Britt Gustafsson, Lars Wallin, Ulrika Winblad, Mio Fredriksson

**Affiliations:** 1 Department of Public Health and Caring Sciences, Health Services Research, Uppsala University, Uppsala, Sweden; 2 Region Dalarna, Dalarna, Sweden; 3 Centre for Clinical Research, Dalarna, Sweden; 4 Department of Health and Welfare, Dalarna University, Falun, Sweden

**Keywords:** Decommissioning, department manager, healthcare, large budget deficits, unit manager

## Abstract

Decommissioning programmes pose a substantial risk of failure compared to other change processes in healthcare. A better understanding of the challenges associated with change processes initiated by resource scarcity faced by healthcare managers is crucial. This study describes and compares department and unit managers’ experiences during the implementation of a large-scale decommissioning programme in a Swedish region. A survey was developed and a cross-sectional study was performed, measuring 172 healthcare managers’ experiences of (1) the region’s leadership, (2) their own participation and (3) their own commitment and responsibility during the implementation of the decommissioning programme. Respondents were 50 department managers and 122 unit managers (93% and 58% response rate, respectively). There was a significant difference between department and unit managers in their experiences of the region’s leadership and their own participation in the decommissioning programme. Unit managers were more dissatisfied with the way it developed compared to department managers. For example, unit managers reported a lower level of leadership support, incentives to participate, and that their knowledge and skills were not fully utilised. Involvement of unit managers in a more fruitful way might enhance the results of decommissioning programmes. This study highlights a key actor in this context: the unit manager.

## Introduction

The role of the healthcare manager is changing rapidly, with extended responsibilities and increasing demands in an overburdened organisation. Faced with challenges such as budget deficits, healthcare managers struggle to manage limited resources, both financial and human.^1^ In many ways they are the link between organisational demands and staff.^2^ To motivate and engage frontline staff is particularly challenging and complex^3^ when implementing reforms or changes that involve savings, setting limits, and adapting and changing accordingly.^4^ Even if well intentioned, these changes can evoke strong feelings among staff, including a sense of loss, disrespect, and powerlessness as previous roles may be replaced or withdrawn.^5-7^ Among the many concepts dealing with resource scarcity and change, decommissioning is described as the planned removal, reduction, and replacement of health services and can lead to a reassessment of existing services and make way for services that are more effective and beneficial to patients.^4,8^ Whatever the change, it requires a great deal of effort from healthcare managers to ensure that it is implemented successfully.^1^

A persistent challenge for healthcare organisations, including those in wealthy countries, is the lack of resources, emerging from demographic changes and technological and medical innovations that drive costs to new and unpredictable levels.^9^ Change processes initiated by resource scarcity in healthcare systems generally occur at many levels in organisations and are addressed not only through cost cuts in staff and materials, but also through different kinds of prioritisations.^10,11^ Priorities made in healthcare departments and units often include decisions about the allocation of resources between diverse types of services, different patient groups, or diverse elements of care. In the long run, issues concerning health equity are often at stake when setting priorities. Decision-making regarding priorities is a demanding task for healthcare managers as decisions must be made based on several rules and guidelines. For example, when allocating resources in Swedish healthcare, decision makers – including department and unit managers – must take into consideration and adhere to the national ethical guidelines for priorities. The guidelines stipulate that a greater proportion of resources should be allocated to the care of those in greater need.^12^ This is a challenging duty, and researchers report on a perceived need for support at all professional levels in the tough process of making priorities due to scarcity of resources.^13^ In addition, the need for incorporating research-based evidence in the decision-making process is another complex issue in the allocation of resources. Some findings suggest that commissioners and managers tend to rely more on local (unofficial) evidence, such as local political priorities and local professional expertise, potentially jeopardising fairness and consistent, effective healthcare.^14^

The risk of change processes failing in healthcare, especially those initiated due to a lack of resources necessitating prioritisation and budget cuts, is reported to be higher compared to other types of changes, such as implementation of medical innovations or guidelines.^4^ Both department managers and unit managers are, among other things, responsible for resource allocation and priorities at the department or unit they lead. This includes the implementation of necessary changes evolving from, for example, a decommissioning programme initiated by politicians and executive leadership teams. Since healthcare managers play a crucial role in supporting and implementing change processes, gaining a deeper understanding of their experiences in managing significant budget deficits can offer important insights regarding how to support, optimise, and leverage the skills of managers to effectively improve healthcare delivery.^2,15^ However, healthcare managers responsibility varies in several ways. In Sweden, for example, the department manager leads the unit managers, and the unit managers are responsible for leading front-line staff. In short, unit managers have a more operational role, focussing on day-to-day activities such as staffing their units and developing nursing care, while department managers have a broader responsibility and strategic perspective, such as healthcare production and patient safety. Potentially, their different roles may lead them to experience financial challenges and attempts to address them through decommissioning programmes in different ways. Studying potential differences could inform strategies for improving the implementation of decommissioning programmes – as the one studied in this case – and provide knowledge to other organisations facing resource constraints or other pressures affecting their sustainability. Therefore, this empirical study investigated department and unit managers’ experiences of a comprehensive structural and organisational change process in one of the regions responsible for funding and providing healthcare in Sweden. It investigated whether there are differences between the 2 groups regarding the experiences of (1) the region’s leadership, (2) their own participation and (3) their own commitment and responsibility.

## Methods

This is a cross-sectional study based on a survey measuring healthcare managers’ experiences of leadership, participation, commitment and responsibility during the process of implementing a decommissioning programme.

### Survey development

To facilitate the work with priority setting, a Canadian research group developed the Resource Allocation Performance Assessment Tool (RAPAT). RAPAT is a battery of items used to identify areas for improvement in terms of priority setting processes within an organisation based on key factors recognised as important for successful prioritisation.^3,16,17^ RAPAT was used as inspiration, and selected items were translated into Swedish, modified, and included in a questionnaire. Another source of inspiration was a survey conducted in Region Västerbotten in Sweden in 2009. The aim of that survey was to explore the employees’ experiences and attitudes to priority setting following a priority setting process in the region’s healthcare organisation.^18,19^ The survey included items that were appropriate for the current study, and after modifying them slightly, they were included in the questionnaire.

Fifteen former department and unit managers assessed the items’ relevance in the first draft of the questionnaire through participating in a content validity survey.^20^ The participants were identified through snowball sampling.^21^ They received instructions on how to assess the relevance of the items to capture and better understand the managers’ function and experience of priority setting and resource allocation during extensive structural and organisational changes. The participants were also asked to verbalise their thoughts about the content and language. This survey and verbal feedback affected 6 items in the questionnaire. One statement with an Item-Content Validity Index (I-CVI) score of 0.67 was deleted, and 5 statements were rephrased. The I-CVI range of the remaining items was 0.87 to 1.0, and the Scale-Content Validity Index (S-CVI) for the total questionnaire was 0.94. A final version with 20 statements was compiled (Appendix 1), and an online questionnaire was designed.

The questionnaire consisted of 3 subscales, each including 6 to 8 items, graded on a five-point Likert response scale (strongly disagree, disagree, neutral, agree, strongly agree) along with the response ‘don’t know’. The first subscale covers the managers’ experiences of the region’s leadership during the change process. The second assesses the managers’ experiences of participation in the change process, and the third measures the participants’ commitment to and responsibility during the change process. Table 1 reports the S-CVI, Cronbach’s alpha and question topics for the 3 subscales of the questionnaire.

**Table 1.** Subscales including S-CVI, Cronbach’s alpha and question topics.

Subscale	S-CVI	Cronbach’s alpha	Question topics
1. Experiences of the region’s leadership during the change process (leadership)	0.95	0.858	Leadership decision-making quality
			Leadership ethical responsibility
			Leadership focus
			Leadership strength
			Leadership support
			Leadership transparency
2. Experiences of participation in the change process (participation)	0.94	0.832	Expression of opinions
			Incentives
			Knowledge use
			Transparency
3. Experiences of commitment and responsibility during the change process (commitment and responsibility)	0.94	0.653	Decision-making quality
			Ethical responsibility
			Implementation commitment
			Loyalty

### Setting

The Swedish healthcare system is strongly decentralised. Each of the 21 politically governed regions in Sweden has its own independent budget and the responsibility to fund and deliver the majority of healthcare services, with approximately 75% of the funding coming from income taxes paid by those residing in the region.

Region Dalarna is the fourth largest region in Sweden in terms of land area, yet it is characterised by a sparse population. About 300 000 inhabitants, 3% of Sweden’s population, live in the region. The low population density in some places is a challenge, and the following issues are always on the agenda in the priority setting discussions: how to ensure (1) accessibility, (2) patient safety and (3) emergency care when healthcare services can be a half-day journey away. In 2015 Region Dalarna had many years of repetitive budget deficits, and the executive leadership of Region Dalarna, represented by politicians and public servants, decided that until 2019, 700 million SEK had to be cut to get the budget in balance. The executive leadership developed a detailed plan, a decommissioning programme. As a start of the decommissioning programme, a comprehensive review was compiled of, for example, the region’s quality of care, costs, staffing, capacity, population and public health, accessibility and effectiveness, as well as revenues. The review-report was focussed on Region Dalarna’s healthcare system but included comparisons with other regions in Sweden. Region Dalarna had high costs and low productivity.^22^ The department managers, but also to some extent unit managers and healthcare professionals, were involved in the decision-making processes of the decommissioning programme by suggesting and evaluating decommissioning proposals to solve the financial problems. A new leadership model was introduced at this time, with a particular focus on the clarification of roles and responsibilities as a department or unit manager. At least short-term, the decommissioning programme did not result in any negative impact on patient safety or quality of care.^23^ Furthermore, the results of the employee survey indicated that the level of satisfaction with the region’s leadership improved.^24^ Almost 95% of the 150 decommissioning decisions were implemented, and in 2020 a national evaluation reported that Region Dalarna had the lowest healthcare cost per inhabitant among Sweden’s 21 regions.^25^

### Sample and data collection

All department and unit managers in Region Dalarna responsible for healthcare services related to patients were invited to participate in the survey. The data collection was carried out through procedures adapted to meet each manager group’s work situation and convenience.

Department managers were invited to participate in the survey during one of their bi-annual department managers’ meetings in April 2018. They were informed about the survey through both the invitation and the agenda for the day. At the meeting, they were informed verbally and in writing about the voluntary nature of the survey and that they could decline participation and not attend the scheduled time for the survey without giving a reason. Fifty managers agreed to participate in the survey and signed a written informed consent form. Four managers were not present at the meeting and did not participate in the study. The overall response rate was 93%.

The unit managers received a web survey distributed through the region’s e-mail system. The e-mail included information about the study’s purpose and the researcher credentials, and it noted that participation was voluntary. By answering the questionnaire, the respondent agreed to participate in the study. In December 2018, a total of 211 unit managers were invited to participate, with 48 initial responders. Three reminder e-mails generated 74 additional responses, resulting in a total number of 122 responses (58% response rate).

The instructions given in the survey stated that regarding department managers, the executive leadership level was defined as the division manager and the executive leadership team. Similarly, in relation to unit managers, the executive leadership level was defined as the department manager, the division manager and the executive leadership team.

Both groups were informed that survey data would only be processed by the researchers and the results would be presented in such a way that no individuals could be identified. The authors had no professional or personal relationship with the healthcare managers who participated in the study. The study was approved by the regional ethics board in Uppsala (Dnr. 2016/504B).

### Analytical procedure

The study included 44 department managers and 93 unit managers. Six department managers and 29 unit managers were excluded from the study on the grounds that they had been present in the decommissioning programme for less than 2 years. Subscale scores were compared between department and unit managers, using the independent-samples *t*-test. The effect size (Cohen’s *d*), mean value, and confidence interval (CI) are presented for the subscales. In addition, the percent of maximum score computed as mean score divided by the subscale’s maximum score is presented for each manager group and subscale. The comparison between department and unit manager for scores of individual items was analysed using the Mann–Whitney *U* test. To investigate whether potential within-group differences could be associated with the managers level of support, question 20 (Overall, I support how the executive leadership team handles priorities and resource allocation) was dichotomised into *disagree* (strongly disagree, disagree and neither) and *agree* (agree and strongly agree) and *t*-tests performed for the 3 subscales.

## Findings

The average number of years in the position of the 137 healthcare managers that were included in the study was 8.1 years (2-38) for department managers 9.2 years (2-32) for unit managers. The questionnaire, results and missing data of all items are presented in Appendix 1.

### Department managers

Taken together, the department managers’ experiences illustrate a rather positive impression of the region’s leadership during the change process (see Tables 2 and 3). This concerned not only perceived support and encouragement from the executive leadership team but also constructive reactions when the department managers questioned the executive leadership’s decisions on priorities and resource allocation. Similarly, almost all department managers felt that the executive leadership team’s leadership was shaped by the common good of the region, rather than by the interests or desires of individuals.

**Table 2.** Comparison of scores between department managers and unit managers.

Subscale	Department manager	% Of max. score	Unit manager	% Of max. score	Mean difference (95% CI)	*P*-value	Cohen’s *d* (95% CI)
1. Leadership (0-40 points)	Mean 25.5 (SD ± 5.2)	64	Mean 20.9 (SD ± 7.3)	52	4.6 (2.1-7.0)	<.001	0.68 (0.31-1.05)
2. Participation (0-30 points)	Mean 23.0 (SD ± 3.9)	77	Mean 17.9 (SD ± 6.3)	60	5.1 (3.0-7.1)	<.001	0.90 (0.53-1.28)
3. Commitment and responsibility (0-30 points)	Mean 24.9 (SD ± 3.1)	83	Mean 23.7 (SD ± 5.1)	79	1.2 (–0.4 to 2.8)	.148	0.27 (–0.09 to 0.63)

*P*-value and confidence interval (CI) based on the independent-samples *t*-test. Percent of maximum score for each manager group and subscale.

**Table 3.** Significant findings on item scourings in subscales (Mann–Whitney *U* test).

Subscale/item	Department manager	Unit manager	*P*-value
1. Leadership			
1. The executive leadership team encourages and supports me as a manager to improve the work with priorities and resource allocation at the unit/department I lead.	4 (0.25)	3 (1)	.002
	n = 44	n = 87	
5. I feel that the executive leadership team reacts constructively if their decisions regarding priorities and resource allocation are questioned by unit/department managers.	4 (1)	3 (1)	< .001
	n = 41	n = 78	
7. The executive leadership teams’ leadership is characterised by the common good of the region, rather than by the interests or desires of individuals.	4 (0)	4 (1)	.012
	n = 43	n = 80	
8. The executive leadership team is good at managing responses from the population to decisions on priorities and resource allocation.	4 (1)	3 (1)	.047
	n = 33	n = 63	
2. Participation			
9. I have the opportunity to express my opinion to the executive leadership team regarding how priorities and resource allocation are handled in Region Dalarna.	4 (1)	3 (2)	< .001
	n = 44	n = 85	
12. There are incentives for me as a manager to participate in the region’s work with priorities and resource allocation.	4 (1)	3 (2)	.005
	n = 43	n = 80	
13. In working with priorities and resource allocation, my skills and experience are put to good use.	4 (1)	3 (2)	.007
	n = 44	n = 86	
14. I know when the different steps in the region’s planning process for the region plan, financial plan, and budget take place.	4 (1)	4 (1)	.037
	n = 44	n = 85	
3. Commitment/Responsibility			
16. At the unit/department I lead, we have made care provision priorities which required reallocation of resources	4 (1)	4 (1)	.048
	n = 44	n = 89	

median, (interquartile range), n = number of respondents.

When turning to the department managers’ experiences of participating in the decommissioning programme, a picture of an inclusive change process emerges. The views of department managers were taken into account; their knowledge and experiences were utilised, and the work with priorities was characterised by transparency.

Even higher scores were observed when the department managers assessed their own commitment and responsibility. They rated their adherence to the executive leadership team’s decisions as very high. In addition, decisions regarding prioritisation and resource allocation in their department were perceived to result in patient groups with the greatest need to a high extent. They also expressed overall support for the executive leadership team’s ability to manage prioritisation and resource allocation during the implementation of the decommissioning programme. However, when dividing the department managers into those who disagreed (n = 7) and those who agreed (n = 34) with the statement ‘Overall, I support how the executive leadership team handles priorities and resource allocation’, there was a significant difference in scorings in all 3 subscales; leadership (*t* = −3.319, *P* = .002), participation (*t* = −3.643, *P* ⩽ .001) and commitment and responsibility (*t* = −3.368, *P* = .002. Agreeing was associated with a higher score.

### Unit managers

The results demonstrate a change process characterised by medium scores regarding the executive leadership team’s leadership and ability to support, encourage, and include unit managers (see Tables 2 and 3). A perceived lack of transparency in decision-making processes, difficulties in questioning decisions made by the executive leadership team, and receiving constructive reactions were other statements that got low scores.

The scores regarding the unit managers’ experiences of being able to participate in the change process tended to be medium. One in 2 unit managers reported few opportunities to express their opinions and also a low degree of perceived transparency and clarity about how priorities and resource allocation were handled by the executive leadership. Likewise, the unit managers reported a low degree of incentives to participate and opportunities to use their skills and experience during the change process.

A different picture emerges when unit managers scored their commitment and responsibility during the change process. The decisions made by the executive leadership team had a direct impact on their units, leading the unit managers to reallocate resources if required. Patient groups with the greatest need were perceived to be prioritised within their units. Although the unit managers mostly implemented the region’s prioritisation decisions, regardless of their personal opinions, the unit managers expressed less positivity about the quality of the data that formed the basis for the decisions. In the same way as for the department managers, when dividing the unit managers into those who disagreed (n = 27) and those who agreed (n = 62) with the statement, ‘Overall, I support how the executive leadership team handles priorities and resource allocation’, there was a significant difference in scorings in all 3 subscales (leadership *t* = −4.808, *P* ⩽ .001), participation (*t* = −2.622, *P* = .010) and commitment and responsibility (*t* = −3.768, *P* ⩽ .001) Also for unit managers agreeing was associated with higher scores.

### Comparing the experiences of department managers and unit managers

Overall, the findings indicate that the 2 groups of healthcare managers had diverse experiences of the implementation of the decommissioning programme and the associated change processes. Unit managers had lower mean scores in all 3 subscales compared to the department managers. The difference was significant for the Leadership subscale as well as the Participation subscale (*P* < .001) (see Table 2). Both manager groups rated their own commitment and responsibility higher than their participation and gave the lowest rating to the region’s leadership during the change process.

The differences in the leadership subscale indicated a medium effect size (Cohen’s *d* = 0.68), while the participation subscale showed a large effect (Cohen’s *d* = 0.90). The commitment and responsibility subscale proved a negligible effect (Cohen’s *d* = 0.27).

Nine of 20 items in the survey showed a significant difference between department and unit managers. Four of these items belonged to the Leadership subscale, and four to the Participation subscale and one to the Commitment and Responsibility scale (see Table 3).

## Discussion

The results of this study clearly demonstrate that the experiences of healthcare managers implementing a large-scale decommissioning programme in a local healthcare organisation differ significantly between unit managers and department managers. While a majority of both department and unit managers expressed support for how the executive leadership team handled the implementation of the decommissioning programme, the results indicate a potential for improvement, highlighting particularly unmet needs among unit managers. Department managers presented higher mean scores in all subscales (Leadership, Participation and Commitment/Responsibility), with a significant difference regarding leadership and participation, and they also seemed to be more satisfied with the process compared to unit managers.

The need for enhanced support from the leadership constitutes an important finding. For example, the unit managers report a lack of support to improve the work with priorities and resource allocation, as well as constructive feedback if questioning the executive leadership team’s decisions. Furthermore, the strength and focus of the executive leadership team were challenged by unit managers, and they reported less confidence in that the leadership is characterised by the region’s common good rather than by the interests or desires of individuals. The latter may indicate an uncertainty about the executive leadership team’s quality of decision-making and could point to a lack of openness and collaboration. Notably, the unit managers’ score for the region’s leadership reached only 52% of the maximum score. The department managers, however, seemed to be less concerned about this issue.

The experiences of unit managers regarding their ability to participate and contribute to the change process clearly indicate that the competence, knowledge, and skills of unit managers were not fully utilised during the implementation of the decommissioning programme. In addition, the unit managers reported a lower degree of incentives to participate in the work with priorities and resource allocation. In line with our findings, scholars have shown that unit managers (middle managers) are an underutilised resource in healthcare organisations with the potential to significantly improve the outcomes of change. For example, unit managers could disseminate, motivate, and adapt change work to make it feasible in time-pressed healthcare organisations. In order to fully implement such important processes, unit managers need support from their managers (in our case, primarily from the department managers) and access to information and knowledge to encourage front-line staff, adapt changes to the daily life of their units, and act as ambassadors in the change processes.^26-29^

Compared to the department managers, the unit managers reported a significantly lower level of opportunity to express their opinions and challenge decisions made by the executive leadership team. Similarly, scholars have reported a feeling of ‘voicelessness’ among first-line managers (unit managers) as they try to cope with complex issues, interests, and unclear mandates between local and central levels in organisations. Difficulties in influencing and participating in decision-making can lead managers to resign.^30^

Department managers are perceived as the primary implementers of change in healthcare, as they can interpret and communicate the rationale and the possible consequences of the change in their departments. To connect decisions and proposals to the real-world context, department managers need to translate, for example, rationales and decommissioning decisions into comprehensible messages for their staff.^2,15,26,31^ In our case, the department managers were explicitly involved in making decommissioning decisions through frequent meetings and collegial discussions. As unit managers experienced a lower level of participation, clarity, and transparency during the change process, this indicates a need for more information and involvement of this group in decision-making about priorities and resource allocation. This may illustrate that department managers need to work closer and more inclusively with their unit managers and secure transparency in decision-making about priorities at their clinics. Findings recommending the involvement of healthcare managers at an early stage in decision-making appear to be a key factor in achieving the objective of reforms, such as decommissioning programmes.^4^ Since healthcare managers have a broad responsibility to prioritise resources without sacrificing patient safety, quality, or ethics, and are ultimately responsible for the outcomes of required decisions, it is important that they are involved in the decision-making process at an early stage.^4,15^ In this particular case, previous research has shown that the reasons for the change, the economic difficulties, were clearly communicated in the organisation and understood by the healthcare managers.^15,32^

By the time the survey was conducted (2018), almost all decommissioning decisions had been implemented. Furthermore, the economy of the region showed clear signs of improvement. Although the unit managers were dissatisfied with the leadership and level of participation, they were committed in their efforts to implement the changes. Perhaps this loyalty is reflected in that the managers reported that the decisions on priorities and resource allocation made by the executive leadership had an impact on the activities they lead, as 91% of the department managers and 80% of the unit managers agreed or strongly agreed with this statement.

Another finding is that only about 50% of both department and unit managers agreed, or strongly agreed, that the decisions made by the executive leadership team lead to the prioritisation of patient groups with the greatest needs. Scholars report that managers demonstrate distress when priorities are made at their clinics in a way that does not harmonise with their responsibility as managers to ensure, for example, patient safety and continuity.^33^ Likewise, nurse managers (often being the unit managers) report a need for more structured discussions and decision-making in ethical problems at clinics and underline the importance of making these problems visible.^34^ In the current study, almost all managers reported that they implemented the regions’ decision regardless of what they personally thought about the decisions. This may either be due to a high level of loyalty or indicate a certain degree of resignation among some managers. As one might have anticipated, the results of the within-group analyses indicate that department and unit managers who expressed support for the executive leadership team’s ability to handle priorities and resource allocation also scored higher in their perception of the region’s leadership, and their own participation, and commitment and responsibility.

In 2023, Region Dalarna projected a deficit of 750 million SEK and decided to implement a new decommissioning programme to balance its finances. This leads to reflection on whether healthcare organisations need to find better ways to deal with resource scarcity. The issue is now being dealt with retroactively in Sweden, such as by implementing decommissioning programmes at regular intervals like every 4 to 5 years. The political leadership’s goals and priorities may quickly change direction and often result in additional requirements about services to be provided within allocated budgets. Other influencing factors may include new technologies and medical developments, such as new effective but expensive drug treatments that require additional funding, which may mean that existing treatments must be reduced or removed. This is a source of stress for all parties involved, not least for managers at various levels who must perform savings and replace, remove, or re-evaluate existing healthcare to meet new requirements. A long-term, careful planning where managers at all levels are involved in a more constructive way, which also takes into account professional requirements, may reduce stress in relation to the demands that healthcare managers face in their daily work.

## Conclusion and Practical Implications

The illustrated differences between department and unit managers highlight potential areas for improvement and clearly emphasises the need for a more effective involvement of unit managers. As decommissioning programmes have proven challenging to implement, conditions will be more conducive if all healthcare staff are genuinely involved. Unit managers play a crucial role in leading front-line staff, thereby motivating and facilitating acceptance of the required changes. During an acute financial crisis, the organisation’s executive leadership team needs to mobilise all healthcare managers to feel and shoulder ownership, responsibility, and accountability in their roles and demonstrate how their efforts contribute to the success of the entire organisation. Healthcare organisations need to address the cost and inefficiencies that arise from healthcare managers experiencing inadequate support or not being able to use their knowledge or skills in such processes.^28^ Leadership support, incentives to participate, and an inclusive decision-making culture might give healthcare managers a fair chance to thrive, develop, and implement savings in an efficient and effective way,^27^ despite the often messy realities of healthcare.

## Limitations and Future Research

Despite a high response rate and most questionnaires being fully completed, this study was limited by missing data (‘do not know’ answers) for some items. This applies particularly to subscale 1 (Leadership) for the unit managers and may indicate that these items were difficult to understand or less relevant for this group of managers. Furthermore, it would have been possible to contact former healthcare managers to ascertain their experiences of the implementation of the decommissioning programme. However, it is unclear whether the responses would still be valid after a period of 5 years. Additionally, the annual turnover rate among approximately 250 healthcare managers was approximately 4%, which is unlikely to have had a significant impact on the outcome. Another limitation is that the study was performed in one region in Sweden, which makes the extent to which the results are generalisable difficult to judge.

Future research should investigate unit managers’ role by focussing on some of our findings. For instance, research could explore how unit managers think about future challenges, such as savings through decommissioning programmes, staff shortages, and patient safety. What do unit managers need in order to cope, thrive, and develop in their work? The unit manager is an important part of the future of healthcare systems, and the need for nursing staff will increase, and by that competent, effective, and passionate unit managers.

## Supplemental Material

sj-docx-1-his-10.1177_11786329241299316 – Supplemental material for Implementing a Decommissioning Programme in Swedish Healthcare: Experiences of Healthcare Managers Supplemental material, sj-docx-1-his-10.1177_11786329241299316 for Implementing a Decommissioning Programme in Swedish Healthcare: Experiences of Healthcare Managers by Inga-Britt Gustafsson, Lars Wallin, Ulrika Winblad and Mio Fredriksson in Health Services Insights
