# Supplementary material for: Implementing a Decommissioning Programme in Swedish Healthcare: Experiences of Healthcare Managers
Source: Health Serv Insights. 2024 Nov 19;17:11786329241299316. doi: 10.1177/11786329241299316 (PMC11577462; doi:10.1177/11786329241299316)

**Appendix 1.**

For *department managers*, the executive leadership level is defined as the division manager and the executive leadership team.

For *unit managers*, the executive leadership level is defined as department manager, division manager, and the executive leadership team.


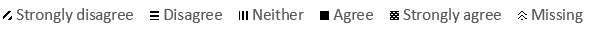


**LEADERSHIP:** Statements about your experiences of the leadership regarding [the work with] priorities and resource allocation in Region Dalarna – strongly disagree, disagree, neither, agree, and strongly agree

***1.*** *The executive leadership team encourages and supports me as a manager to improve the work with priorities and resource allocation at the unit/department I lead. (p=0.002)*


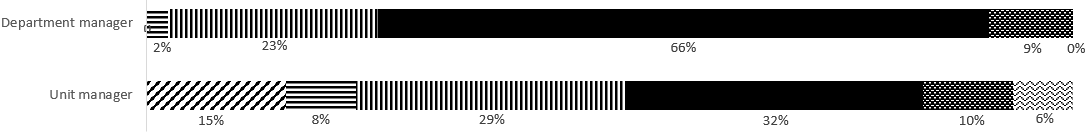


***2.*** *The decisions on priorities and resource allocation made by the executive leadership team lead to patient groups with the greatest need being prioritised.* *(p=0.889)*

**
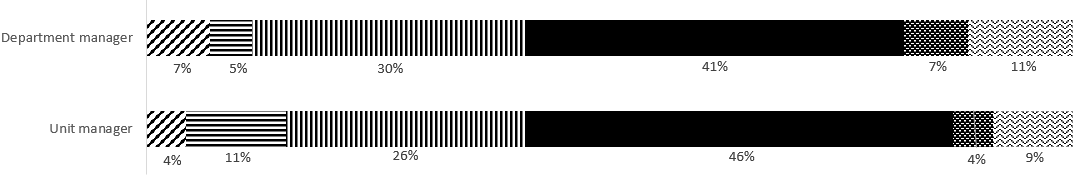
**

***3.*** *The material/data that inform priorities and resource allocation within my division are of high quality. (p=0.174)*


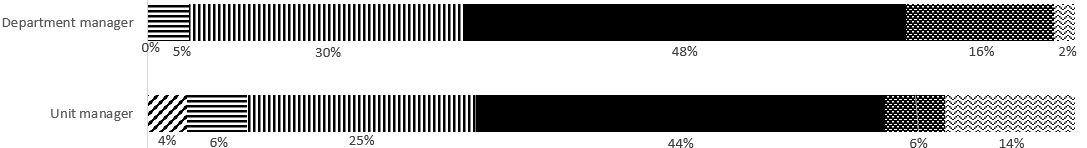


***4.*** *Agreements regarding priorities and resource allocation in Region Dalarna are characterised by transparency and clarity. (p=0.142)*

*
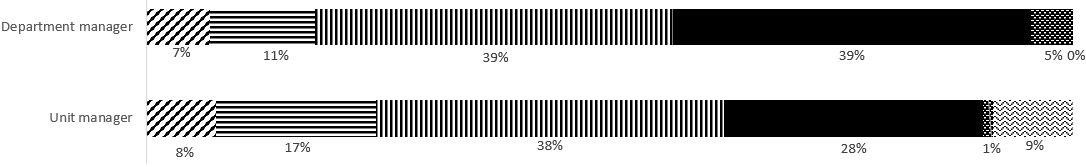
*

***5.*** *I feel that the executive leadership team reacts constructively if their decisions regarding priorities and resource allocation are questioned by unit/department managers.* *(p=< .001)*


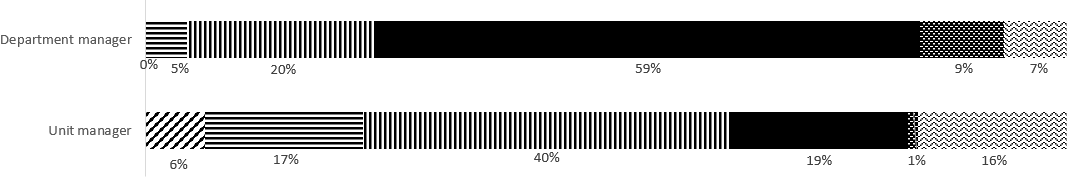


***6.*** *I feel that the executive leadership team takes reasonable measures against unit or department managers who oppose or do not participate in the region's work with priorities and resource allocation. (p=0.134)*

*
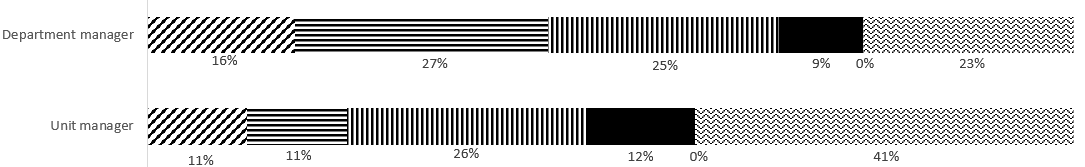
*

***7.*** *The executive leadership teams' leadership is characterised by the common good of the region, rather than by the interests or desires of individuals. (p=0.012)*

*
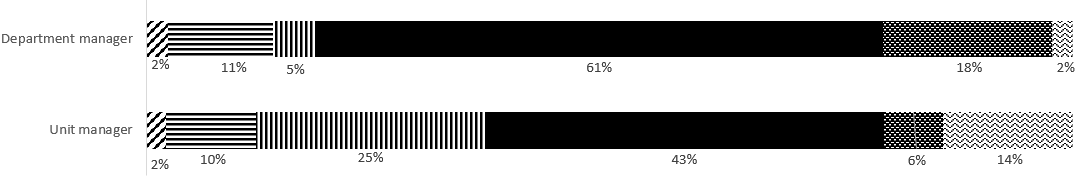
*

***8.*** *The executive leadership team is good at managing responses from the population to decisions on priorities and resource allocation. (p=0.047)*

**
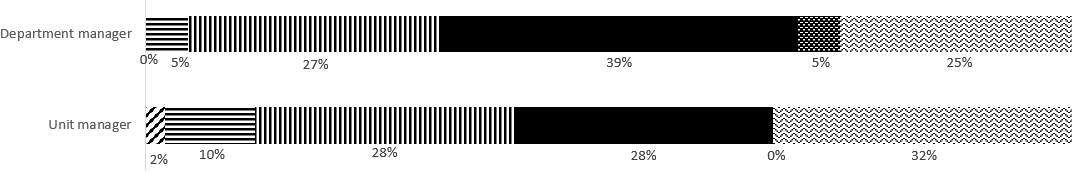
**


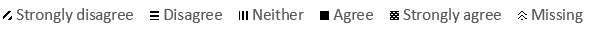


**PARTICIPATION:** Statements about your participation in the work with priorities and resource allocation – strongly disagree, disagree, neither agree, and strongly agree

***9.*** *I have the opportunity to express my opinion to the executive leadership team regarding how priorities and resource allocation are handled in Region Dalarna. (p=< .001)*

**
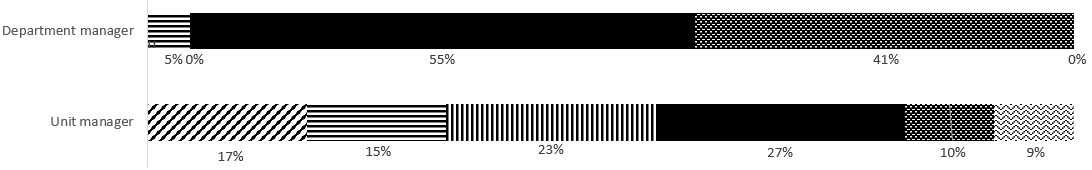
**

***10.*** *The work with priorities and resource allocation at the unit/department I lead is characterised by transparency and clarity. (p=0.098)*


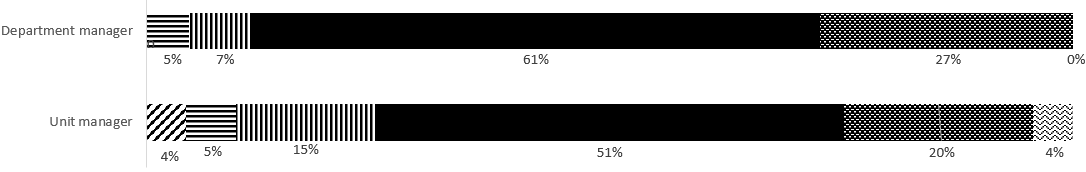


***11*.** *The work with priorities and resource allocation in divisions and departments is characterised by transparency and clarity. (p=0.321)*


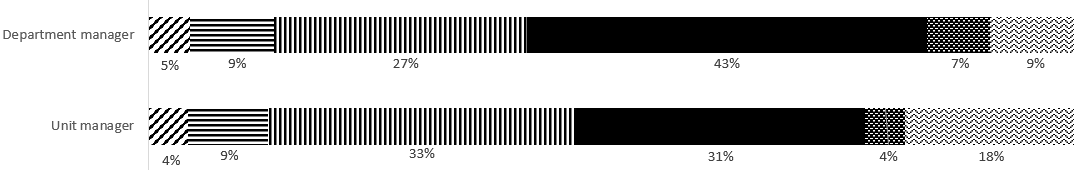


***12.*** *There are incentives for me as a manager to participate in the region's work with priorities and resource allocation. (p=0.005)*


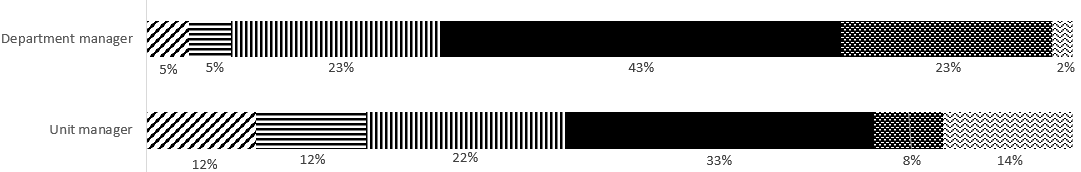


***13*.** *In working with priorities and resource allocation, my skills and experience are put to good use.*

*(p=0.007)*


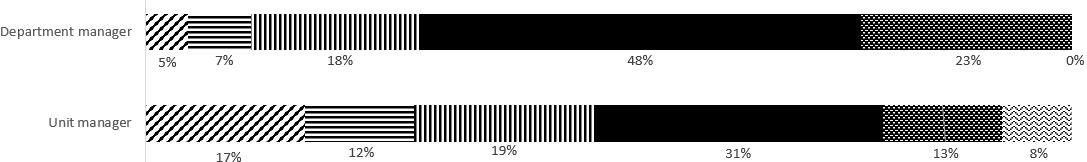


***14.*** *I know when the different steps in the region's planning process for the region plan, financial plan, and budget take place. (p=0.037)*


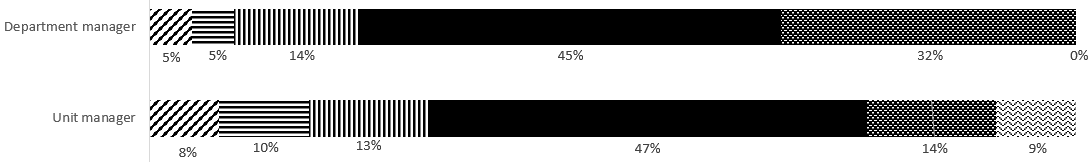


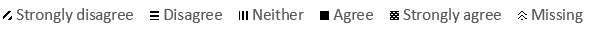


**COMMITMENT/RESPONSIBILITY:** Statements about your commitment and responsibility in the work with priorities and resource allocation – strongly disagree, disagree, neither, agree, and strongly agree

***15.*** *I contribute to ensuring that the decisions on priorities and resource allocation made by the executive leadership have an impact on the activities I lead. (p=0.107)*


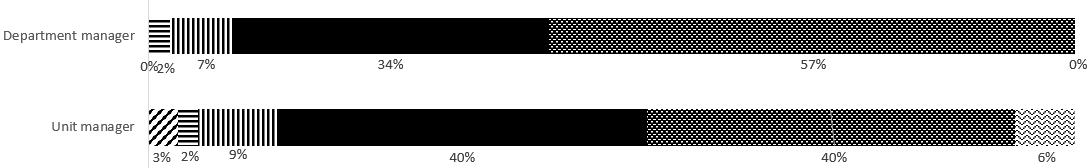


***16****. At the unit/department I lead, we have made care provision priorities which required reallocation of resources. (p= 0.048)*


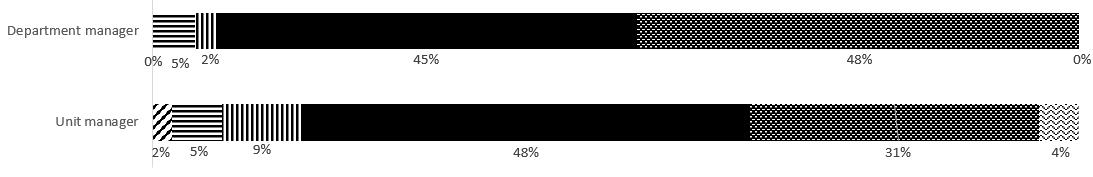


***17.*** *The material/data that inform priorities and resource allocation at the unit/department I lead are of high quality. (p=0.559)*

**
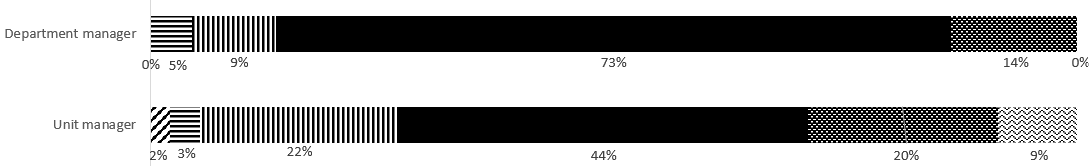
**

***18.*** *The decisions about priorities and resource allocation made at the unit/department I lead result in patient groups with the greatest need being prioritised. (p=0.175)*


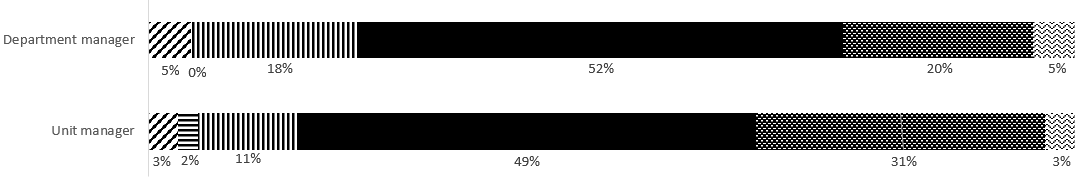


***19.*** *As manager, I implement the regions' decisions regarding priorities and resource allocation, regardless of what I personally think about the decisions. (p= 0.424)*

**
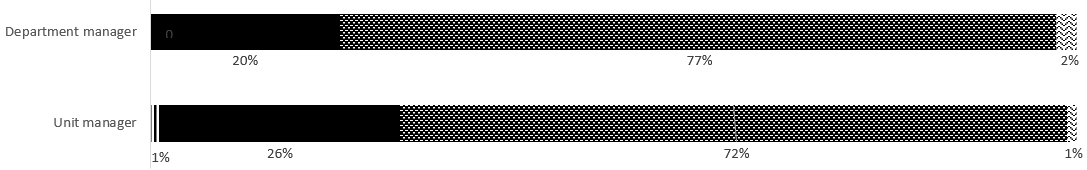
**

***20.*** *Overall, I support how the executive leadership team handles priorities and resource allocation.*

*(p= 0.731)*


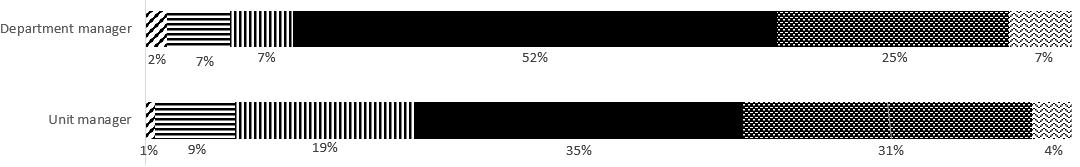

Supplement: sj-docx-1-his-10.1177_11786329241299316 – Supplemental material for Implementing a Decommissioning Programme in Swedish Healthcare: Experiences of Healthcare Managers [file sj-docx-1-his-10.1177_11786329241299316.docx]
